# Development of cardiac remodeling in patients with acute myocardial infarction studied by cardiac MRI (CMR)

**DOI:** 10.1186/1532-429X-17-S1-P130

**Published:** 2015-02-03

**Authors:** Martin R Sinn, Enver Tahir, Ulf K Radunski, Dennis Säring, Maxim Avanesov, Kai Muellerleile, Gerhard Adam, Gunnar Lund

**Affiliations:** Department of Diagnostic and Interventional Radiology, University Medical Center Hamburg-Eppendorf, Hamburg, Germany, Hamburg, Germany; Departments of Cardiology, University Medical Center Hamburg-Eppendorf, Hamburg, Germany; Department of of Computational Neuroscience, University Medical Center Hamburg-Eppendorf, Hamburg, Germany, Hamburg, Germany

## Background

Left ventricular (LV) remodeling after acute myocardial infarction (AMI) is defined as a progressive increase of end-diastolic volume (EDV) ≥ 20% compared to baseline (BL). However, the exact occurrence of LV remodeling and the further development of EDV after AMI is unclear.

## Methods

LV myocardial mass, volumes, and function were assessed in 25 patients after reperfused first AMI using cine and delayed enhancement imaging . Patients underwent CMR within 7±4.9 days (baseline, BL) after AMI. Follow-up scans were performed at 1.25±0.27 months (FU1), 3.3±0.56 months (FU2) and 6.27±0.65 months (FU3).

## Results

Two types of LV remodeling were observed. Three of 25 patients (12%) (age: 55±7 years) showed normal EDV at baseline with 166 ±12 ml, but significantly increased EDV with 219 ±32 ml at FU1(*P*<0.01 vs BL). At FU2 and FU3 no further changes in EDV were detected in these patients (Table 1). Another three patients (12%, age: 45±8 years) showed early remodeling with significantly increased EDV of >200 ml at BL and no further change of EDV during follow-up (Table 1). Infarct size was significantly higher in the group of patients with remodeling compared to patients without remodeling (Table 1). At BL, patients with early remodeling had a significantly lower ejection fraction (EF) with 33±5% compared to patients with no remodeling (56±9%, *P*<0.01). At FU3, patients with early remodeling show recovery in function, whereas patients with delayed remodeling showed no change in EF during follow-up (Table 1).

**Table 1:** Data are presented as mean±SD of patients.

		BL	FU1	FU2	FU3
**EDV [ml]**	No remodeling (n=19)	143±27	156±31	152±28	150±32
	Delayed remodeling (n=3)	166±12^†^	219±32^*#^	209±58^*^	211±32^*^
	Early remodeling (n=3)	251±44^*^	246±20^*^	240±27^*^	247±34^*^
**Infarct size [% of LV]**	No remodeling (n=19)	12±8	11±8	10±8	9±7
	Delayed remodeling (n=3)	25±7^*^	26±8^*^	25±6^*^	23±8^*^
	Early remodeling (n=3)	22±8^*^	19±9^*^	16±12^*^	20±7^*^
**EF [%]**	No remodeling (n=19)	56±9	58±8	58±7	61±8
	Delayed remodeling (n=3)	47±11	44±16	46±18	45±11
	Early remodeling (n=3)	33±5^*^	37±2^*^	39±7^*^	46±7^*^

* P<0.01 vs no remodeling

# P<0.05 vs BL

† P = ns vs no remodeling

## Conclusions

Remodeling occurred in 6 of 25 patients (24%) after AMI. Three patients (12%) showed early remodeling with increased EDV and low EF already at BL, which improved at 6 months follow- up. In another three patients (12%) delayed remodeling occurred at FU1 with stable EDV and EF at later follow-up. Remodeling occurs in patients with large infarction either early with initially increased EDV and or delayed with initially normal EDV. Early remodeling is characterized by initially severely reduced EF with recovery in EF at 6 months follow-up.

## Funding

This study is partially funded by the Deutsche Forschungsgemeinschaft.

